# Highly pathogenic avian influenza A virus subtype H5N1 (clade 2.3.4.4b) isolated from a natural protected area in Peru

**DOI:** 10.1128/mra.00417-24

**Published:** 2024-08-16

**Authors:** Sandra Landazabal-Castillo, Dilan Suarez-Agüero, Lucero Alva-Alvarez, Enrique Mamani-Zapana, Egma Mayta-Huatuco

**Affiliations:** 1 National University of San Marcos, Molecular and Clinical Virology Laboratory, Lima, Peru; DOE Joint Genome Institute, Berkeley, California, USA

**Keywords:** Influenza A viruses, H5N1, birds, Peru

## Abstract

The panzootic caused by H5N1 avian influenza viruses is a high concern for wild birds’ conservation and the study of spillover events into mammals. The near coding-complete genome of H5N1 clade 2.3.3.4b sequencing in the Miseq Illumina platform was performed from a bird located in Pantanos of Villa National Wildlife Refuge.

## ANNOUNCEMENT

Highly pathogenic avian Influenza (HPAI) H5N1 virus, a member of the family *Orthomyxoviridae* and genus *AlphaInfluenzavirus*, has reemerged globally in multiple outbreaks of rapid distribution since 2020 and has caused alarming mortality rates in wild birds. The H5N1 strains emerging during the last years have also been associated with mass mortality of poultry and mammals in Peru and worldwide (1–5).

An oro-pharyngeal swab from a live Calidris alba bird (6), exhibiting lethargy and difficulty to fly in Pantanos of Villa Wildlife Refuge (Lima, Peru), was collected, in April 2023, an inactivating viral transport media was used. The sample was processed in the Molecular and Clinical Virology Laboratory of the National University of San Marcos, biosecurity level II (BSL-2). First, the RNA was extracted using the Viral Nucleic acid extraction kit II Geneaid, and cDNA synthesis was performed through RevertAid First-Strand cDNA Synthesis Kit ThermoFisher following the manufacturer’s protocols. Then, avian influenza virus (AIV) detection was done using high-resolution melting analysis (HRM) to target the M and H gene (7), by a MIC PCR magnetic induction cycler of Biomolecular Systems (BMS). A subsequent end point PCR assay was run using Mytaq Red DNA Polymerase kit Meridian Bioscience using MBTuni-12 and MBTuni-13 primers (8, 9) and thermocycling conditions set up as follows: 3 min at 95°C, then 5 cycles of 30 s at 95°C, 30 s at 45°C, 3 min at 68°C, followed by 35 cycles of 30 s at 95°C, 30 s at 57°C, 3 min at 72°C, with a final extension at 72°C for 5 min. Finally, DNA bands were excised and purified using NucleoSpin Gel and PCR Clean-up Macherey Nagel. Libraries were also generated for amplicon-based sequencing using Nextera XT DNA Library preparation kit Illumina (10), which were pooled in equimolar concentrations and underwent the Miseq Reagent v2 chemistry (250-cycle paired-end) on the Miseq platform (Illumina) according to the manufacturer’s instructions (11).

**Fig 1:**
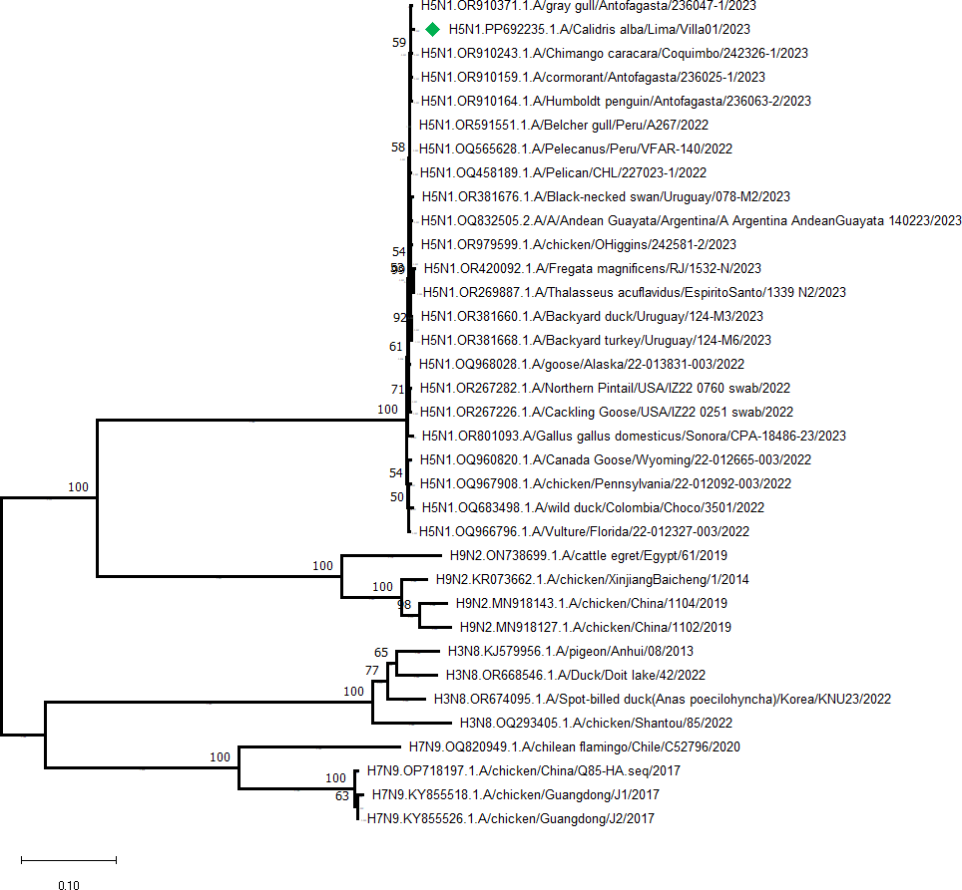
Phylogenetic tree based on complete sequences of HA gene. The neighbor-joining method was used, with 1,000 bootstrap replicates, Kimura-2 parameters (K2P) model, and gamma distribution. The analysis included 34 nucleotide sequences. Isolate H5N1.A/Calidris alba/Lima/Villa01/2023 is highlighted in green.

Default parameters were used for all software unless otherwise noted. In total, 79,377 raw sequencing reads were obtained, with a mean length of 251 nucleotides per read. Data trimming was done using Trimmomatic v.039 (12), and clean reads were *de novo* assembled using Megahit v.1.2.9 (13). The sequences obtained had an average coverage of 1,161×, a length of 747–2,322 bp, and the GC content was 44.75%. Eight segments of IAV were identified using BLASTn analysis of NCBI database (14), showing high homology (99.55%–99.86%) with modern South America AIV (Table 1). Consensus sequences were aligned by MAFFT 7.526 (15) and the neighbor-joining phylogenetic tree of the HA gene segment was performed by MEGA v.11 (16) (Fig. 1). The determination of mutations was carried out using the web application Flusurver (17).

**TABLE 1:** BLAST comparison of nucleotide sequences of eight segments of isolate H5N1.A/Calidris alba/Lima/Villa01/2023 with those closely related strains

Gene segment	Length (bp)	% GC	Depth of coverage (x)	Most closely related strain	Identity (%)	Reference sequence accession number GenBank
PB2	2322	45	6.13	A/pelican/Peru/PIUSER013/2022(H5N1)	99.61	OQ925705.1
PB1^a^	1783	42.7	64.93	A/guanaycormorant/Tarapaca/236301/2023(H5N1)	99.61	OR960991.1
PA	2263	43.6	13.42	A/pelican/Peru/PIU-SER019/2022(H5N1)	99.55	OQ550449.1
HA	1757	41.7	349.49	A/graygull/Antofagasta/236047-1/2023(H5N1)	99.60	OR910371.1
NP	1565	48.1	1623.22	A/Guanay cormorant/Peru/PIUSER024/2022(H5N1)	99.81	OQ550427.1
NA	1458	43.9	229.13	A/gray gull/Chile/C61947/2022(H5N1)	99.86	OQ352558.1
M^a^	747	48.5	2846.10	A/Peruvian booby/Coquimbo/239024/2023(H5N1)	99.60	OR979582.1
NEP	865	44.5	4160.57	A/chicken/Araucania/241914-1/2023(H5N1)	99.65	OR125217.1

^a^ Partial coding sequence; all others listed are complete coding sequences.

H5N1 HPAIV virus clade 2.3.4.4b was identified from viral isolate H5N1.A/Calidris alba/Lima/Villa01/2023 with a cleavage site in PLREKRRKGLF and the presence of deletion mutations (K343del and R344del) (18, 19). However, molecular markers associated with mammalian adaptation were not detected, nonetheless the presence of (S149A) mutation in HA (20, 21), this finding sheds light on the virus’s evolving affinity for host receptors and its transmissibility, by a decreased ability to bind α2,3-SA and increased to bind α2,6-SA; thus, it indicates a zoonotic potential of the virus for the chance of host-specific shift (22–25).

Influenza viruses must be studied upon One Health approach to monitor viral evolution and acquired mutations such as those associated with mammalian adaptation. Moreover, these viruses must be analyzed by a multisegmented approach to have better epidemiological and molecular surveillance.

## Data Availability

The eight obtained segments were deposited in GenBank (accession numbers PP692232-39). The raw sequence reads were deposited under SRA accession numbers SRR28478279/85. The sequences were also deposited in EpiFlu at GISAID (EPI3229624-31).

## AFTER EPUB

*[This article was published on 16 August 2024 with errors in the keywords. The keywords were corrected in the current version, posted on 23 August 2024.]*
